# Pregnancy in Morrison Pouch: a case report

**DOI:** 10.11604/pamj.2024.47.95.42627

**Published:** 2024-02-29

**Authors:** Frank Martin Sudai, Lusajo Mwagobele, Lameck Mdengo, Rabson Bagoka, Stanley Zakaria Binagi, Joseph Bee, Jesca Paul Lebba

**Affiliations:** 1 Ministry of Health, Maweni Regional Referral Hospital, Kigoma, Tanzania,; 2 President´s Office, Region Authorities and Local Governments, Region Health Management Team, Kigoma, Tanzania

**Keywords:** Abdominal pregnancy, ectopic pregnancy, maternal hemorrhage, case report

## Abstract

Abdominal pregnancy is a rare form of ectopic pregnancy (accounting for 1% of all ectopic pregnancies). Depending on gestational age and its location various symptoms and signs may be exhibited. This study aimed to report a case of abdominal pregnancy occurring in the Morrison Pouch with a primary presentation of right upper quadrant pain and to highlight complications that may arise in the management of abdominal pregnancy located in the Morrison Pouch. A 22-year pregnant woman at gestation of 22 weeks presented with a right upper quadrant mass and pain. Ultrasound examination revealed a live extrauterine singleton at Morrison Pouch, full blood count showed severe anemia. The patient received a blood transfusion in seven days and underwent emergency laparotomy after experiencing sudden acute internal hemorrhage but died a few hours post laparotomy due to hemorrhagic shock. Abdominal pregnancy carries a high risk of maternal hemorrhage as described in this case.

## Introduction

Abdominal pregnancy is a rare occurrence approximately 1% of all ectopic pregnancies where a fertilized ovum implants outside the uterus either primarily or secondarily and it accounts for 1 in 10,000 live births [1]. The common implantation site for abdominal pregnancy is the pelvic peritoneum followed by uterine serosa, bowels, and omentum; the rarest occurrence is in the liver, spleen, and retroperitoneal [2].

## Patient and observation

**Patient information:** a 22-year-old female G2P0+1L0 at 22 weeks gestational age by date who presented with dull right upper quadrant pain and a palpable mass. The pain was not radiating to the back, and there was no associated jaundice or history of fever. She had a spontaneous abortion at 8 weeks a year ago with no complications, no history of contraceptive use, and is not yet booked for antenatal visits.

**Clinical findings:** the patient appeared conscious, pallor, and afebrile with normal vital signs. A palpable mass measuring approximately 16cm by 12cm was detected on the right upper quadrant but it was non-tender on palpation.

**Timeline of the current episode:** on 9^th^ July 2023; admitted, laboratory investigations and ultrasound done. At 01 am on 15^th^ July 2023, the patient fainted, was hypotensive with internal bleeding, and was taken for emergency explorative laparotomy. At 09 am on 15^th^ July 2023, the patient passed away three (3) hours post the laparotomy.

**Diagnostic assessment:** on admission laboratory investigations; full blood count revealed severe anemia with a hemoglobin level of 6.5g/dl and MCV 67fl, blood group O positive, urine pregnancy test positive, abdominopelvic ultrasound showed alive singleton extrauterine fetus in Morrison´s Pouch at a gestational age of 23 weeks, with neither hematoma nor features of internal bleeding. The patient did not perform MRI due to the unavailability of the exam.

**Diagnosis:** on admission advanced abdominal pregnancy at a gestational age of 22 weeks with severe anemia was established. But after seven days she succumbed to a diagnosis of acute internal bleeding secondary to abdominal pregnancy.

**Figure 1:**
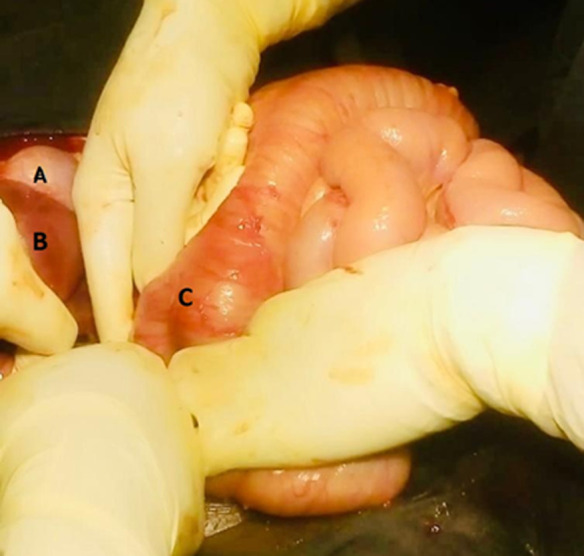
A) intraoperative findings before delivery of the baby, gall bladder; (B) right lobe of the liver; (C) and proximal third of the transverse colon

**Figure 2:**
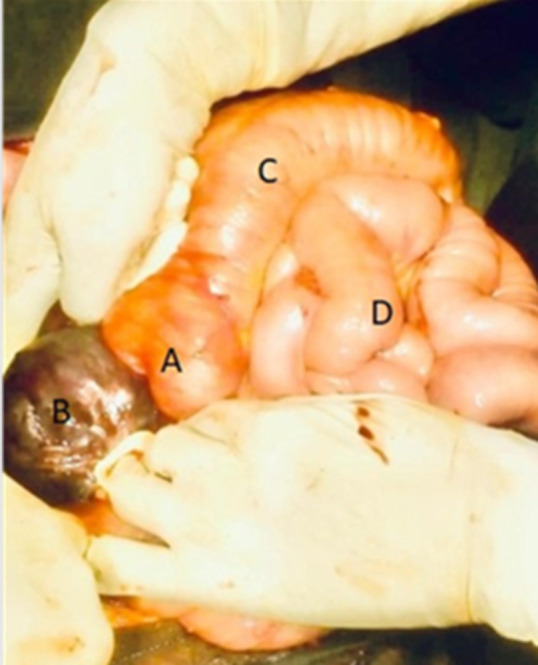
A) intraoperative findings: proximal third of transverse colon (hepatic flexure); (B) head of the fetus; (C) mid-third of transverse colon; (D) and small bowel

**Figure 3:**
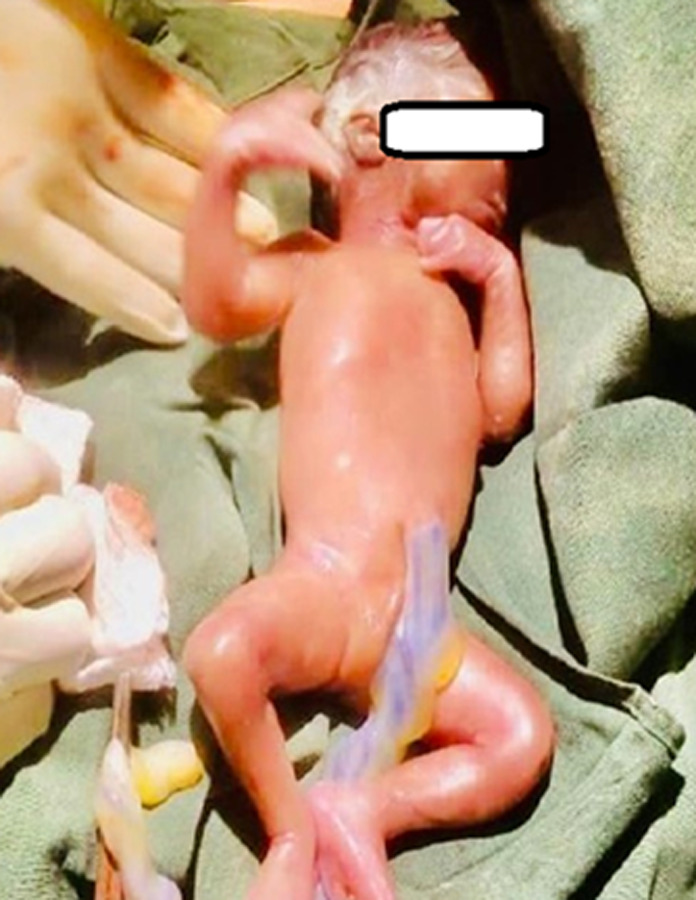
the fetus soon after delivery who scored 4 on the first and 0 on the fifth minute

**Therapeutic interventions:** four units of whole blood were transfused on alternating days with ferrous sulfate and folic acid (205mg) once daily. On the seventh day post-admission, the patient suddenly experienced acute internal bleeding necessitating an emergency explorative laparotomy where an extended mid-line incision was made and intraoperative 2 liters of hematoma and fresh blood evacuated. The hepatic flexure of the transverse colon was identified and clear visualizations of the liver and gall bladder were obtained (Figure 1), the fetus' head was observed at the lower aspect of the right lobe (Figure 2). However, it was noted that there was no presence of an amniotic sac, indicating that it had already ruptured. A male baby weighing 500mg with an Apgar score of 4 at the 1^st^ minute and 0 at the 5^th^ minute was extracted (Figure 3). Following delivery, there was significant bleeding as the placenta detached spontaneously from the inferior aspect of the right lobe of the liver. Hemostatic packs were applied to the affected area where the placenta had been implanted, and hemostasis was achieved with difficulty. As a precautionary measure, abdominal drainage was placed in situ. The abdomen was closed in layers. The total estimated blood loss during the procedure was 3 liters and she received 3 units of whole blood intra-operatively. Vitals after the procedure were as follows; BP=105/69mmHg, PR=108bpm, spo_2_=89% on mechanical ventilation.

**Follow-up and outcome of interventions:** postoperative the patient was admitted to ICU and placed on mechanical ventilation, another unit of blood and antibiotics were administered, however 3 hours postoperative, the abdominal drainage collected 1 liter of fresh blood and oxygen saturation dropped from 89%. During admission to 40%, BP=59/37mmHg, PR=118bpm. Resuscitation measures were initiated while in ventilation by 1 liter of normal saline, chest compression, and three cycles of adrenaline shots but after 30 minutes, attempts to stabilize the patient were unsuccessful, death was certified and the cause of death was hemorrhagic shock.

**Patient perspective:** clinically patient progress deteriorated post-operative and resuscitation was done with no success. The patient´s relative was grateful for the efforts made by the team participating during treatment regardless of the outcome.

**Informed consent:** we obtained consent from the relative for the write-up and publication of this case.

## Discussion

Abdominal pregnancy can be categorized as either primary or secondary. When the embryo directly implants into the abdominal cavity it is considered the primary type, on the other hand, secondary abdominal pregnancy happens as a result of complications such as a ruptured tubal pregnancy, tubal abortion, uterine rupture, or perforation. Furthermore, in the management approach, abdominal pregnancy can be classified as early (at or below 20 weeks gestational age) or advanced when gestational age at diagnosis is above 20 weeks [3]. The natural history of an advanced abdominal pregnancy has a majority of cases (80%) with maternal hemorrhage requiring blood transfusion and fetal mortality of up to 72% [4]. Symptoms and signs may be absent for an early abdominal pregnancy but symptoms are much expected with the advanced type and these include but are not limited to palpable fetal parts, abdominal pain as in our case, and fetal growth restriction [3]. The diagnosis can be made by an ultrasound and an MRI can be adjunctly used to accurately locate the placenta and blood supply in relation to the surrounding structures.

The early diagnosis of this rare condition was made promptly upon admission, which was a considerable strength in managing this complex case. Early detection allows for timely decision-making, minimizing the risk of complications such as hemorrhagic shock [5]. Identifying this condition promptly enables healthcare professionals to provide tailored treatment plans and initiate early intervention, potentially improving patient outcomes. The management of this case suffered from delayed intervention. Delayed intervention can lead to a range of complications, including hemorrhagic shock, as we observed in this case. The delay may have been due to challenges in surgical planning for abdominal pregnancies.

It is crucial to emphasize the significance of timely management in cases of abdominal pregnancy with approaches such as surgical exploration, evacuation of the abdominal pregnancy, and control of bleeding to greatly minimize the risk of complications like hemorrhagic shock. Early intervention, coupled with a multidisciplinary approach involving obstetricians, surgeons, and anesthesiologists, is essential to optimize patient outcomes [6].

## Conclusion

The diagnosis of ectopic pregnancy at Morrison Pouch is a rare case in our settings and specialties. This case report emphasizes the importance of considering abdominal pregnancy as a potential diagnosis in reproductive age group women presenting with right upper quadrant pain. It underscores the necessity of multidisciplinary approach, including obstetricians, surgeons and anesthesiologists in managing such complex cases. Furthermore, it highlights the significance of early identification and intervention to mitigate potential life-threatening complications.
